# Psychometric properties of the Malay-version beck anxiety inventory among adolescent students in Malaysia

**DOI:** 10.3389/fpsyt.2022.989079

**Published:** 2023-01-25

**Authors:** Nur Husna Ismail, Nik Ruzyanei Nik Jaafar, Luke Sy-Cherng Woon, Manisah Mohd Ali, Rahima Dahlan, Aimi Nur Athira Putri Baharuddin

**Affiliations:** ^1^Department of Psychiatry, Faculty of Medicine, Universiti Kebangsaan Malaysia, Kuala Lumpur, Malaysia; ^2^Faculty of Social Science and Humanities, Center for Research in Psychology and Human Wellbeing, Universiti Kebangsaan Malaysia, Bangi, Selangor, Malaysia; ^3^Department of Psychiatry, Faculty of Medicine and Health Sciences, Universiti Putra Malaysia, Serdang, Selangor, Malaysia

**Keywords:** adolescents, anxiety, factor analysis, psychometric, screening, Malaysia, validation study

## Abstract

**Background:**

The Beck Anxiety Inventory (BAI) is a common tool for screening anxiety symptoms. In Malaysia, the Malay-version 21-item BAI has been previously validated in the Malaysian adult population. However, information regarding its reliability and validity among adolescents below 18 years old is still lacking. The objective of this study is to investigate the psychometric properties of the Malay-version BAI in this population.

**Methods:**

The Malay versions of the BAI and the Depression, Anxiety, and Stress Scale (DASS) were administered among a sample of lower secondary school students (*n* = 329, age range: 13–14 years) in Selangor, Malaysia. Cronbach's alpha value for the internal consistency of the Malay-version BAI was determined. The correlation coefficient between the BAI score and DASS anxiety subscale score was calculated to examine convergent validity. The factor structure of the Malay-version BAI was identified by exploratory factor analysis (EFA) using principal axis factoring.

**Results:**

The study included 329 respondents, who were predominantly female (58.7%) and Malay (79.9%). The mean Malay-version BAI score was 14.46 (SD = 12.39). The Malay-version BAI showed a high level of internal consistency (Cronbach's alpha = 0.948) and convergent validity with the DASS anxiety subscale score (r = 0.80, *p* < 0.001). The EFA suggested a one-factor solution, with the factor loading of all items on the single factor ranging between 0.48 and 0.81.

**Conclusion:**

The Malay-version BAI demonstrated good psychometric properties. It can be a valid and reliable screening instrument for anxiety among Malaysian adolescents.

## Introduction

Anxiety disorders are among the most common mental health issues in the adolescent population. Surveys showed many adolescents experienced anxiety and required treatment for anxiety disorder (1). According to the World Mental Health Surveys, only 41.3% of the global population meeting the criteria for an anxiety disorder thought they needed care (2). Anxiety disorders among adolescents also cause an increased risk for suicidal behavior (3). In a study by Windarwati et al. (4) among 869 high school adolescents, 72.7% of teens experienced anxiety in the mild to very severe categories. The study also showed a significant relationship between suicidal ideation with anxiety in adolescents. The Malaysian National Health and Morbidity Survey (NHMS) (5) reveals that the mental health condition of adolescents is increasingly worrisome, with many teenagers aged 13 to 17 years old found to be suffering from mental health problems. One in five people experienced depression (18.3%), two in five people suffered from anxiety symptoms (39.7%), and about one-tenth experienced stress (9.6%). There was also an increasing trend for the prevalence of suicidal ideation to 10% compared to 7.9% in 2012.

In Malaysia, a study that looked at the trends of mental health problems among children and adolescents based on three population-based surveys by Ahmad et al. (6) found that the prevalence of mental health problems among children and adolescents aged 5 through 15 years showed an increasing trend from 19.4% in 2006 and 20% in 2011. Studies were also conducted among young adolescents aged 13 to 17 whereby at this stage they experienced physical and/or emotional changes, and depression was one of the most common mental health problems that could occur among them.

Adolescents with anxiety problems experience considerable impact on their learning such as loss of interest in learning and personality changes such as lethargy, low self-confidence or lack of self-esteem. According to Nguyen et al. (7), 19.4% of students in secondary school with low self-esteem were detected at a prevalence of related to anxiety, depression, educational stress, and suicidal ideation. Hamid et al. (8) reported a moderate level of depression, anxiety, and stress level for a total of 270 teenage students. The authors suggested that those who suffered from depression, anxiety, and stress problems to be given clinical attention and mitigated before becoming worse among students.

According to Hein et al. (9) the most utilized method for evaluating the presence of anxiety is by self-reporting questionnaires. This method is easy to administer because respondents would only have to answer a list of related questions found in the inventory provided. Among the questionnaires to measure anxiety is Beck's Anxiety Inventory (BAI). BAI, created by Aaron T. Beck and colleagues, is a self-report inventory consisting of 21 items that measure the severity of anxiety among adults and adolescents. Since items in BAI describe emotional, physiological, and cognitive symptoms of anxiety but not depression, it can discriminate anxiety from depression. Even though the instrument was initially applied in the age range of 17–80 years old, it has also been used in peer-reviewed studies with younger adolescents aged 12 and older (10).

Two early studies examined the use of the BAI among adolescents. The study by Kumar et al. (11) in an adolescent psychiatric inpatient sample (aged 12–17 years) in Philadelphia, United States found that the BAI displayed a high level of internal consistency (Cronbach's alpha = 0.91). In another study by Steer et al. (12) among 105 outpatients aged between 13 and 17 years at the same center, the BAI again demonstrated excellent internal consistency, with a Cronbach's alpha value of 0.92. In a separate study among adolescent inpatients, the BAI also displayed high internal consistency (Cronbach's alpha = 0.94) and convergent validity with the Revised Children's Manifest Anxiety Scale (13).

Various factor structures of BAI have been found. Beck et al. (14) and Hewitt and Norton (15) supported similar two-factor models corresponding to cognitive and somatic dimensions of anxiety. These factors showed good internal consistency and test-retest reliability. Subsequently, Beck and Steer (16) identified four factors in the BAI reflecting subjective, neurophysiological, autonomic, and panic components of anxiety. Borden et al. (17) preferred a five-factor solution consisting of subjective fear, somatic nervousness, neurophysiological, muscular/motoric, and respiration. Specifically, for adolescent populations, both Kumar et al. (11) and Steer et al. (12) favored a two-factor solution, representing subjective and somatic symptoms of anxiety, respectively.

Mukhtar and Zulkefly (18) investigated the factor structure, reliability, and validity of the Malay-version BAI among Malaysian adults with a large sample of study participants (*n* = 1,090) with an age range of 18 years to 63 years. In their exploratory factor analysis, they found a three-factor solution (subjective anxiety, autonomic, and neurophysiology) for the BAI, which accounted for 48.01% of the total variance. The BAI also demonstrated good internal consistency (Cronbach alpha coefficient = 0.91).

Previous studies have demonstrated the original BAI as a reliable and valid screening instrument for anxiety symptoms across different demographic sections, including among adolescents. It has been successfully translated into the Malay version, which has also shown good psychometric properties. However, the validity and reliability of the Malay version BAI have yet to be examined among Malaysian adolescents. Furthermore, the BAI has displayed various factor structures in different populations. Therefore, in this study, we aimed to examine the psychometric properties of the Malay version of BAI (Malay-BAI) among adolescents in Malaysia.

## Methods

### Study design

This study was a part of a larger research project that aimed to explore the role of social-emotional learning in improving mental wellbeing among Malaysian adolescents. The current study was conducted to support the development and validation of the learning module. It was a cross-sectional survey of lower secondary school students. Four schools were selected in the state of Selangor, Malaysia. Convenience sampling was carried out to select the schools and recruited the participants in this study.

### Participants

The inclusion criteria were as follows: (1) first-year and second-year secondary school students aged 13 and 14 years old; (2) students of government day schools; (3) provided informed consent; (4) able to comprehend the Malay language questionnaires. Students who underwent psychiatric/ psychological treatment were excluded from this study. This was ascertained by a simple screening question i.e. “Have you received psychiatric/ psychological treatment in the past?” in the demographic section of the questionnaire with 2 options, “Yes” or “No.” Using a subject-to-item ratio of 1:10 (19, 20), the minimum sample size required for the validation of the Malay version BAI was 210.

### Instruments

The questionnaire used in this study contained three sections: demographic data, the Malay-BAI, and the Malay version Depression, Anxiety, and Stress Scale (Malay-DASS). The questionnaire was administered in the Malay language as Malay is the national language of Malaysia. Therefore, all students in Malaysian public secondary schools, including those from minority ethnic groups such as Chinese and Indians, possess basic proficiency in the language, which allowed the universal application of the Malay-language questionnaire in this study. Nevertheless, in a multi-ethnic country like Malaysia, methods of socio-culture adaptation should be considered particularly in adapting health status measures like BAI as highlighted by Beaton et al. (21).

#### Demographic data

Participants filled up a demographic data sheet that covered information on the personal background of participants (age, gender, race, and year of study) and their parents (parent occupation, parent academic achievement, total parental income, and marital status).

#### Malay-version beck anxiety inventory

The measure contains 21 items on anxiety symptoms. The symptoms were rated on a four-point scale. Scores range from 0 to 63. A total score of 0 to 7 is considered minimal anxiety in range, 8–15 is mild anxiety, 16–25 is moderate anxiety, and 26–63 is severe anxiety (22, 23). Mukhtar and Zulkefly (18) first validated the Malay-version BAI for the Malaysian adult population using back-translating procedures by a team of content and linguistic experts whereby in the process, any word ambiguity and colloquial differences were resolved for the overall suitability.

#### Malay version of the depression anxiety stress scale

The instrument contains 21 items with three self-report scales designed to measure the emotional states of depression, anxiety, and stress. It was validated in the Malaysian population with Cronbach's alpha coefficients of 0.75 (depression), 0.74 (anxiety), and 0.79 (stress) by (24). The scale ranges from “did not apply to me at all” (0) to “applied to me very much, or most of the time” (3). Subjects responded to the items and the sub-score of the anxiety component was used to compare with the BAI. The summed score of the DASS anxiety sub-scale (items 2, 4, 7, 9, 15, 19, 20) was used in this study.

### Study procedure

After obtaining informed consent from the parents of study participants, the researchers received the contact information of the students from the school counselors from each school involved in this survey. The researchers then distributed the link to the online survey form containing the demographic data section, Malay-BAI, and Malay-DASS to the participants *via* email and text messages. Data collection was performed through live video conferencing in five sessions to assist participants in answering the questionnaires. Data collection lasted from August until October 2021 during the pandemic COVID-19. It took approximately 30 to 45 min in total to complete the data collection process. This duration included time to brief the participants about the study procedure, explain how to answer the questions, and wait for the participants to submit the questionnaire. The actual time needed by the participants to answer the questionnaire was approximately 10 to 20 min.

### Ethics approval

Ethics approval was obtained from the Research Ethics Committee of The National University of Malaysia (Approval No.: UKM PPI/111/8/JEP-2021-182).

### Statistical analysis

In this study, the Statistical Package for the Social Sciences (IBM-SPSS^®^) version 26.0 software was used for data analyses. Data were checked for completeness. The normality of continuous data was checked using the Kolmogorov-Smirnov test. Descriptive statistics were generated for the sociodemographic variables and the scores of the Malay-BAI and the Malay-DASS anxiety subscales, using numbers and percentages for categorical variables, and medians and interquartile ranges for the non-normally distributed continuous variables. Cronbach's alpha was calculated for the internal consistency of the Malay-version BAI. Spearman's correlation coefficient between the BAI score and the DASS anxiety subscale score was calculated to examine convergent validity. The factor structure of the Malay-version BAI was identified by exploratory factor analysis (EFA) using principal axis factoring. All 21 items of the BAI were subjected to the analysis. The Kaiser-Meyer-Olkin (KMO) test was used to verify sampling adequacy for the analysis, while Bartlett's test was used to check the suitability of the correlation structure for factor analysis. A parallel analysis was conducted to determine the number of factors to retain, using an online parallel analysis engine to generate random eigenvalues (25).

## Results

### Descriptive statistics

A total of 329 participants responded to this study. Demographic characteristics are shown in Table 1. The age was between 13 years old (48.3%) and 14 years old (51.7%); 58.7% (*n* = 193) were female and 41.3% (*n* = 136) were male. Malays formed the majority (61.7%). The median score for the Malay-BAI was 11.0 (IQR = 16.5), while the median score for the Malay-DASS anxiety sub-score was 5.0 (IQR = 6.5).

**Table 1:** Socio-demographic of 329 respondents.

**Variable**	**No. of respondents**	**%**
**Age (year)**		
13	159	48.3
14	170	51.7
**Gender**		
Male	136	41.3
Female	193	58.7
**Race**		
Malay	263	79.9
Chinese	8	2.4
Indian	56	17.0
Other	2	0.6
**Year of study (lower secondary)**		
First year	159	48.3
Second year	170	51.7

### Internal consistency and convergent validity

The 21-item Malay-BAI displayed excellent internal consistency with a calculated Cronbach's alpha value of 0.95. The Spearmen's correlation coefficient between the Malay-DASS anxiety subscale and Malay-BAI indicated a strong positive correlation (Spearman's Rho = 0.80) between the two measures, which was statistically significant (*p* < 0.001). This statistically significant strong relationship between the Malay-BAI score and the Malay-DASS anxiety subscale score demonstrated convergent validity between these two measurements of the same construct, namely anxiety symptoms among the study participants. Meanwhile, the Malay-BAI score also displayed a significant correlation with both the Malay-DASS depression subscale (Spearman's Rho = 0.72, *p* < 0.001) and stress subscale (Spearman's Rho = 0.75, *p* < 0.001), but with slightly lower values for the correlation coefficients.

### Exploratory factor analysis

The results of the KMO measure of sampling adequacy (=0.959) and Bartlett's Test of Sphericity (*p* < 0.001) indicated that the data was suitable for factorial analysis (26). Inspection of the correlation matrix revealed no evidence of multicollinearity as none of the bivariate correlation coefficients were > 0.80 (27). Furthermore, all items had a measure of sampling adequacy of above 0.80, which provided a good indication that the items were appropriate for inclusion in the factor analysis (26). Using the parallel analysis approach, the eigenvalues computed from the data were compared against randomly generated eigenvalues. In the comparison, only the first eigenvalue based on the original data (10.546) was greater than the random eigenvalue (1.478), while the second eigenvalue (1.334) was already smaller than the next random eigenvalue (1.391). Thus, the number of eigenvalues was one (28). This suggested a one-factor solution to the Malay-BAI. Since there was only a single factor, no rotation was performed. As shown in Table 2, the factor loadings of all BAI items on the single factor ranged from the lowest of 0.48 (Item 19, “Faint / lightheaded”) to the highest of 0.81 (Item 11, “Nervous”). Meanwhile, the range of value for communality was between 0.36 (Item 16, “Fear of dying”) and 0.72 (Item 3, “Wobbliness in legs” and Item 5, “Fear of worst happening”).

**Table 2:** Factor loading and communality for the single-factor solution for the 21-item BAI (*N* = 329).

		**Factor loading**	
		**1**	**Communality**
1.	Rasa kebas *Numbness or tingling*	0.61	0.52
2.	Rasa panas *Feeling hot*	0.63	0.55
3.	Jalan terhuyung hayang *Wobbliness in legs*	0.71	0.72
4.	Sukar untuk bertenang/relaks *Unable to relax*	0.73	0.59
5.	Takut sesuatu yang buruk akan berlaku *Fear of worst happening*	0.79	0.72
6.	Kepala pusing atau berat *Dizzy or lightheaded*	0.79	0.70
7.	Debaran jantung meningkat *Heart pounding/racing*	0.77	0.65
8.	Rasa tidak stabil/tidak stabil *Unsteady*	0.73	0.63
9.	Cemas *Terrified or afraid*	0.79	0.67
10.	Rasa amat takut *Nervous*	0.81	0.70.
11.	Rasa tercekik *Feeling of choking*	0.69	0.61
12.	Tangan ketar *Handstrembling*	0.72	0.52
13.	Rasa bergoyang *Shaky / unsteady*	0.78	0.70
14.	Takut akan hilang kawalan *Fear of losing control*	0.72	0.53
15.	Sesak nafas *Difficulty in breathing*	0.68	0.54
16.	Takut seperti ingin mati *Fear of dying*	0.51	0.36
17.	Takut *Scared*	0.80	0.71
18.	Perut tidak selesa *Indigestion*	0.78	0.68
19.	Pengsan *Faint / lightheaded*	0.48	0.54
20.	Muka menjadi kemerahan *Face flushed*	0.57	0.61
21.	Berpeluh bukan kerana panas *Hot/cold sweats*	0.68	0.64

## Discussion

The purpose of this study was to investigate the psychometric properties of the Malay version of the BAI among Malaysian adolescent students. In this study sample, the Malay-BAI has demonstrated excellent internal consistency with a Cronbach's of 0.95 and displayed good convergent validity with the anxiety subscale of the Malay-DASS. The EFA suggested a single-factor structure for the Malay-BAI.

The median score for the Malay-BAI in this study was 11.0 (IQR = 16.5). In a study by Osman et al. (10) in an adolescent inpatient sample, the mean BAI score was 15.5 (SD = 12.7). This was comparable to the mean BAI score (15.7, SD = 14.8) reported by Jolly et al. (13) in another study among adolescent inpatients. Similarly, the mean BAI score in an outpatient adolescent study was 16.0 (SD = 12.6) (12). The average BAI score in the current study was lower likely due to the community sample involved in contrast to the clinical samples in the cited studies. When compared with the finding from the validation study of the Persian version of the BAI among adolescents (mean = 8.0, SD = 6.9), our sample indicated a higher average level of anxiety (29). Unfortunately, the average BAI score for Malaysian adults in the community is unavailable for comparison, as the only known study utilizing the Malay-BAI (18) did not report the value.

Concurring with previous research on the use of the BAI among adolescents, our study found that the BAI displayed excellent internal consistency when administered to Malaysian adolescents. The Cronbach alpha value (0.95) in the current study was better than the earlier validation of the Malay-BAI among adults (0.91) (18). Moreover, the Malay-BAI has shown very good convergent validity with the anxiety subscale of the Malay-version DASS scale, an instrument that has been validated in adolescents (30). These findings suggest that the Malay translation of the BAI, which was originally intended for use among adult respondents, can be reliably administered to younger adolescents. There was no indication that the language and content of the instrument were hard for the study participants to comprehend and respond to appropriately.

The single-factor model of the BAI identified in this study is different from most of the factor structures of the BAI found in the existing literature. Various two-factor, four-factor, and five-factor models have been reported (11, 12, 14–17). The Malaysian adult study on the Malay-BAI proposed a 3-factor structure (subjective anxiety, autonomic, and neurophysiology factors) (18). The study by Osman et al. (10) among adolescent inpatients initially identified a four-factor solution. Nevertheless, their final analysis supported a single-factor structure, similar to the current study. The different factor models of the BAI could probably be explained by the heterogeneity of the study population, differences in culture and language between Malaysia and western countries, and different understanding of anxiety symptoms between adults and adolescents. Even so, the unidimensional model of the Malay-BAI in this study does support its use as a measure of anxiety as a unitary construct among adolescents.

There were several limitations to this study. Since no diagnostic interview for clinical anxiety disorders was included, no reference standard could be used to determine the concurrent validity, sensitivity, and specificity of the Malay-BAI. Likewise, the cut-off score for clinical anxiety using the Malay-BAI could not be decided in this sample by building a ROC curve. As this study included non-Malay participants to be representative of Malaysia's diverse cultures, another possible study limitation was the lack of cross-cultural adaptation. Subtle cultural differences might necessitate further adaptations before the Malay-BAI is administered to individuals of different ethnic backgrounds despite the common understanding of the Malay language, to ensure content validity across cultures. This could be considered in future research. The generalizability of the study findings to entire Malaysia might also be limited by the convenience sampling method and sampling in the highly urbanized Selangor state alone. Nonetheless, the demographic profile of our study sample contained a good mix of both genders and the main ethnic groups in the country, thus allowing wider application of the study results to the Malaysian adolescent population to some extent. Despite these limitations, to the authors' knowledge, the present study is the first to investigate the psychometric properties of the Malay-BAI in the range age below 18 years. Future research should strive to further address the need for valid and reliable identification of Malaysian adolescents with anxiety, including but not limited to a confirmatory factor analysis to further verify the factor structure of the Malay-BAI.

## Conclusion

The Malay version of BAI has demonstrated excellent internal consistency and convergent validity when employed among Malaysian adolescents. It also appeared to measure anxiety as a single dimension in this sample. Findings from this study suggest that the Malay-version BAI can be a suitable instrument to screen for anxiety in this population.

## Data availability statement

The raw data supporting the conclusions of this article will be made available by the authors, without undue reservation.

## Ethics statement

The studies involving human participants were reviewed and approved by Research Ethics Committee of The National University of Malaysia (Approval No.: UKM PPI/111/8/JEP-2021-182). Written informed consent to participate in this study was provided by the participants' legal guardian/next of kin.

## Author contributions

NI, NN, and LW: conceptualization and methodology. NI and AB: data curation. NI and LW: formal analysis. NI: project administration, resources, and writing-original draft. LW, NN, MM, and RD: supervision. NN: validation. LW and NN: writing-review and editing. All authors have read agreed to the published version of the manuscript.

## References

[1] Blanco C Rubio J Wall M Wang S Jiu CJ Kendler KS. Risk factors for anxiety disorders: common and specific effects in a national sample. Depress Anxiety. (2014) 31:756–64. 10.1002/da.22247 24577934 PMC4147018

[2] Alonso J Liu Z Evans-Lacko S Sadikova E Sampson N Chatterji S. World mental health surveys in 21 countries. Depress Anxiety. (2018) 35:1–24. 10.1002/da.22711 29356216 PMC6008788

[3] Beesdo K Bittner A Pine DS Stein MB Höfler M Lieb R. Incidence of social anxiety disorder and the consistent risk for secondary depression in the first three decades of life. Arch Gen Psychiatry. (2007) 64:903–12. 10.1001/archpsyc.64.8.903 17679635

[4] Windarwati HD Lestari R Wicaksono SA Kusumawati MW Ati NAL Ilmy SK. Relationship between stress, anxiety, and depression with suicidal ideation in adolescents. Jurnal Ners. (2022) 17:36–41. 10.20473/jn.v17i1.31216

[5] Institute for Public Health. National Health and Morbidity Survey 2017 (NHMS 2017): Adolescent Health Survey. Ministry of Health Malaysia (2017).

[6] Ahmad N Muhd Yusoff F Ratnasingam S Mohamed F Nasir NH Mohd Sallehuddin S. Trends and factors associated with mental health problems among children and adolescents in Malaysia. Int J Cult Ment Health. (2015) 8:125–36. 10.1080/17542863.2014.907326 26000035 PMC4409054

[7] Nguyen DT Wright EP Dedding C Pham TT Bunders J. Low self-esteem and its association with anxiety, depression, and suicidal ideation in Vietnamese secondary school students: a cross-sectional study. Front Psychiatry. (2019) 10:698. 10.3389/fpsyt.2019.00698 31611825 PMC6777005

[8] Hamid R Ghani MFA Ali SKS Daud MAKM Dewi R. (2021). Kemurungan, Kebimbangan Dan Tekanan Dalam Kalangan Pelajar Tingkatan Empat Di Daerah Kota Setar. JuPiDi: Jurnal Kepimpinan Pendidikan. 7:30–4.

[9] Hein D Mai C Hussmann H Maximilian L. The usage of presence measurements in research: a review. In: Proceedings of the International Society for Presence Research Annual Conference (Presence). Prague: The International Society for Presence Research. (2018).

[10] Osman A Hoffman J Barrios FX Kopper BA Breitenstein JL Hahn SK. Factor structure, reliability, and validity of the beck anxiety inventory in adolescent psychiatric inpatients. J Clin Psychol. (2002) 58:443–56. 10.1002/jclp.1154 11920696

[11] Kumar G Steer RA Beck AT. Factor structure of the beck anxiety inventory with adolescent psychiatry inpatients. Anxiety Stress Coping. (1993) 6:125–31. 10.1080/1061580930824837411920696

[12] Steer RA Kumar G Ranieri WF Beck AT. Use of the beck anxiety inventory with adolescent psychiatric outpatients. Psychol Rep. (1995) 76:459–65. 10.2466/pr0.1995.76.2.459 7667457

[13] Jolly J Arruffo J Wherry J Livingston R. The utility of the beck anxiety inventory with inpatient adolescents. J Anxiety Disord. (1993) 7:95–106. 10.1016/0887-6185(93)90008-9

[14] Beck AT Epstein N Brown G Steer RA. An inventory for measuring clinical anxiety: psychometric properties. J Consult Clin Psychol. (1988) 56:893–7. 3204199 10.1037//0022-006x.56.6.893

[15] Hewitt PL Norton GR. The beck anxiety inventory: a psychometric analysis. Psychol Assess. (1993) 5:408–12. 10.1037/1040-3590.5.4.40822121998

[16] Beck AT Steer RA. Relationship between the beck anxiety inventory and the hamilton anxiety rating scale with anxious outpatients. J Anxiety Disord. (1991) 5:213–23.

[17] Borden JW Peterson DR Jackson EA. The beck anxiety inventory in nonclinical samples: Initial psychometric properties. J Psychopathol Behav Assess. (1991) 13:345–57.

[18] Mukhtar F Zulkefly NS. The beck anxiety inventory for Malays (BAI-Malay): a preliminary study on psychometric properties. Malaysian J Med Health Scie. (2011) 7:75–81. 10.7275/jyj1-4868

[19] Costello AB Osborne J. Best practices in exploratory factor analysis: four recommendations for getting the most from your analysis. Pract Assess Res Eval. (2005) 10:1–9.

[20] Ali Memon M Ting H Cheah J-H Thurasamy R Chuah F Huei Cham T. Sample size for survey research: review and recommendations. J Appl Struct Equ Modell. (2020) 4:2590–4221.

[21] Beaton DE Bombardier C Guillemin F Ferraz MB. Guidelines for the process of cross-cultural adaptation of self-report measures. Spine. (2000) 25:3186–91. 11124735 10.1097/00007632-200012150-00014

[22] Beck AT Steer RA. Manual for the Beck Anxiety Inventory. San Antonio, TX: Psychological Corporation (1990).

[23] Steer R Beck A. The Beck Anxiety. Lanham, MD: Scarecrow (1997).

[24] Ramli M Salmiah M Nurul Ain M. validation and psychometric properties of Bahasa Malaysia version of the depression anxiety and stress scales (DASS) among diabetic patients. Malaysian J Psychiatry. (2009) 8:1–7.

[25] Vivek PH, Singh, SN, Mishra, S, Donavan, DT,. Parallel Analysis Engine to Aid in Determining Number of Factors to Retain using R [Computer software]. (2017). Available online at: https://analytics.gonzaga.edu/parallelengine/ (accessed October 21, 2021).

[26] Kaiser HF Rice J. Little jiffy mark IV. Educ Psychol Meas. (1974) 34:111–7. 10.1177/001316447403400115

[27] Field A. Discovering Statistics Using IBM SPSS Statistics. 5th ed. Los Angeles: Sage. (2018).

[28] Horn JL. A rationale and test for the number of factors in factor analysis. Psychometrika. (1965) 30:179–85. 10.1007/BF02289447 14306381

[29] Khesht Masjedi M Omar Z Masoleh S. Psychometrics properties of the persian version of beck anxiety inventory in North of Iranian adolescents. Int J Educ Psychol Res. (2015) 1:145–53. 10.4103/2395-2296.152233

[30] Hashim HA Golok F Ali R. Factorial validity and internal consistency of Malaysian adapted Depression Anxiety Stress Scale-21 in an adolescent sample. Int J Collab Res Intern Med Publ Health. (2011) 3.

